# Optimizing Fetal Surveillance in Fetal Growth Restriction: A Narrative Review of the Role of the Computerized Cardiotocographic Assessment

**DOI:** 10.3390/jcm14197010

**Published:** 2025-10-03

**Authors:** Bianca Mihaela Danciu, Anca Angela Simionescu

**Affiliations:** 1 Department of Obstetrics and Gynecology, Carol Davila University of Medicine and Pharmacy, 050474 Bucharest, Romania; 2 Department of Obstetrics, Gynecology and Neonatology, National Institute for Maternal and Child Health “Alfred Rusescu”-Polizu, 127715 Bucharest, Romania; 3 Department of Obstetrics and Gynecology, Filantropia Clinical Hospital, 050474 Bucharest, Romania

**Keywords:** antepartum, cardiotocography, fetal growth restriction, fetal surveillance, heart rate, short term variation, perinatal outcome

## Abstract

**Background/Objectives:** Fetal growth restriction (FGR) is a leading cause of perinatal morbidity and mortality. Accurate surveillance and timely delivery are critical to improving outcomes. This narrative review examines the role of computerized cardiotocography (cCTG) and short-term variation (STV) interpretation in the monitoring of FGR and its integration with Doppler velocimetry and the biophysical profile (BPP). **Methods:** A comprehensive literature search of PubMed, Scopus, and Web of Science was performed for studies published up to 2021 using combinations of terms related to FGR, CTG, STV, and Doppler surveillance. Eligible sources included original studies, systematic reviews, and international guidelines. Case reports, intrapartum-only monitoring, and studies involving major anomalies were excluded. **Results:** Reduced STV consistently correlates with fetal compromise, abnormal Doppler findings, and adverse perinatal outcomes. In early-onset FGR (<32 weeks), ductus venosus abnormalities often coincide with or precede STV reduction; combined use supports optimal timing of delivery. In late-onset FGR (≥32 weeks), STV changes are less pronounced and require integration with cerebroplacental ratio, variability indices, and trend-based interpretation. Longitudinal evaluation offers greater prognostic value than isolated measurements. However, heterogeneity in thresholds, fragmented outcome data, and system-specific definitions limit standardization and comparability across studies. **Conclusions:** cCTG provides an objective and adjunct to Doppler and BPP in the surveillance of FGR, a tool for obstetrician needs. Its greatest utility lies in serial, integrated assessment, supported by gestational age-specific reference ranges. Future advances should include standardized STV thresholds, large outcome-linked databases, and artificial intelligence-driven tools to refine decision-making and optimize delivery timing.

## 1. Introduction

Fetal growth restriction (FGR, intrauterine growth restriction) represents one of the most prevalent complications of pregnancy, with an incidence ranging from 3% to 9% of pregnancies in high-income countries and reaching up to 25% of pregnancies in low- and middle-income nations [1]. FGR is not only a leading cause of perinatal morbidity and mortality but also contributes to long-term adverse outcomes, including cardiovascular disease, chronic kidney disease, cognitive impairment, and metabolic syndrome [2,3,4]. Preterm birth (<37 weeks) exacerbates the association between FGR and adverse neurologic outcomes. Prematurity is thus a confounding factor when studying the long-term developmental impact of FGR [4].

Diagnosing FGR based solely on an estimated fetal weight (EFW) below the 10th percentile is inaccurate, as it does not effectively differentiate between fetuses that are constitutionally small but healthy, classified as small for gestational age (SGA), and those that are truly growth-restricted due to pathological conditions like intrauterine hypoxia and starvation. Additionally, this criterion may overlook fetuses with an EFW above the 10th percentile who nonetheless exhibit signs of impaired growth and are at risk of adverse outcomes [5].

Additionally, a lot of babies with a fetal size less than the 10th percentile are constitutionally small and healthy, whereas FGR is a pathologic condition. Also, the accuracy of fetal weight estimation may be undermeasured by abdominal circumference or the Hadlock 3 formula used to EFW may be not precise (deviation +/− at term of 500 g) [6].

In order to minimize the risk of false-positive and false-negative diagnoses of FGR, a Delphi consensus definition, predominantly developed by European clinicians, proposes a combined assessment of fetal size (including EFW and abdominal circumference) and abnormal Doppler parameters in the umbilical, uterine, and middle cerebral arteries as presented in Table 1. Despite its clinical utility, the implementation of this definition is constrained by the absence of standardized guidance on which fetal growth chart should be employed to determine the 10th and 3rd percentiles for EFW and abdominal circumference.

Perinatal morbidity and mortality rates are markedly elevated in fetuses with an EFW below the third percentile. Accordingly, some experts have advocated for a revision of diagnostic criteria to classify these fetuses as growth restricted. However, this proposed threshold has not yet been formally incorporated into current clinical guidelines [8].

### 1.1. Etiology and Pathophysiology of Fetal Growth Restriction

Placental insufficiency is the leading cause of FGR, though other factors may also impair fetal growth. For example, genetic anomalies account for approximately 5% of FGR cases [9]. Maternal conditions such as chronic hypertension, systemic lupus erythematosus, antiphospholipid syndrome, gestational or pregestational diabetes mellitus, and other cardiopulmonary or renal diseases have been identified as significant contributors. Furthermore, maternal exposure to substances including cocaine, alcohol, nicotine, marijuana, heroin, antineoplastic agents, or radiation can adversely affect fetal growth. Additional maternal factors include uterine malformations, the use of assisted reproductive technologies, chronic antepartum hemorrhage, and residence at high altitude. These diverse etiologies underscore the multifactorial nature of FGR and the importance of comprehensive maternal and fetal evaluation.

The neuroendocrine axis plays a crucial role in fetal development, and its dysfunction can significantly contribute to the onset of FGR. One of the key mechanisms involves fetal insulin deficiency, which impairs the uptake and utilization of nutrients, directly affecting intrauterine growth [10]. Insulin-like Growth Factor I (*IGF-I*) is a major regulator of somatic growth and cellular proliferation. In addition, it facilitates the transport of amino acids and glucose across the placenta [11]. Low levels of IGF-I have been strongly associated with significant growth restriction. Other growth-related factors—such as Insulin-like Growth Factor II (*IGF-II*), Insulin-like Growth Factor Binding amniotic fluid Protein 2 (*IGFBP-2*), Insulin-like Growth Factor Binding Protein 3 (*IGFBP-3*) and vasoactive intestinal peptide (*VIP*)—have also been implicated in the complex regulation of fetal growth [12].

Thyroid hormone imbalances can further influence fetal development. Hyperthyroidism may lead to decreased oxygen consumption and reduced glucose oxidation, thus lowering the energy available for growth. Conversely, hypothyroidism has been shown to decrease circulating IGF-I levels, compounding the effects of growth restriction [13].

Another important endocrine marker is pregnancy-associated plasma protein-A (*PAPP-A*), secreted by the decidua into the maternal circulation. PAPP-A degrades Insulin-like Growth Factor Binding Protein 4 (*IGFBP-4*), which in turn increases the bioavailability of *IGFs*. Low maternal levels of *PAPP-A* in the first trimester have been identified as a potential early marker for FGR [14].

These findings highlight the importance of assessing the neuroendocrine and growth factor profiles in pregnancies at risk for FGR, as they may provide valuable insights.

### 1.2. Clinical Classification and Diagnostic Challenges

Fetal growth restriction is typically divided into two categories: early-onset FGR (<32 weeks) and late onset FGR (≥32 weeks).

Early-onset FGR is generally more readily identified through ultrasound evaluation. In cases resulting from placental insufficiency, fetal deterioration tends to follow a relatively predictable sequence of Doppler alterations, beginning with abnormalities in the umbilical artery and progressing to changes in the DV. The primary clinical challenge in these cases lies in perinatal management—specifically, determining the optimal mode and timing of delivery—while balancing the competing risks of stillbirth and extreme prematurity [15].

In contrast, late-onset FGR—typically occurring at term or near term—is more difficult to diagnose, as it is often associated with normal Doppler findings in both the umbilical artery and DV. In such cases, fetal weight estimations must be interpreted in the context of the longitudinal growth trajectory rather than as isolated values. The progression of fetal compromise is less predictable, and the assessment of cerebroplacental ratio near term presents interpretative challenges. Moreover, there is a persistent risk of sudden fetal deterioration and intrauterine death despite seemingly reassuring surveillance parameters [15].

The evaluation of fetuses affected by FGR is of critical importance, as fetal developmental disturbances cannot be compensated for later in life and may profoundly alter the newborn’s long-term prognosis. An ideal fetal assessment should integrate Doppler velocimetry with biometric measurements and CTG monitoring parameters.

The International Federation of Gynecology and Obstetrics (FIGO) recommends that when classic biometric measurements indicate an EFW below the 10th percentile, further evaluation should be undertaken to determine whether true FGR is present [16]. Routine ultrasound evaluation in the third trimester is not proved to improve prenatal outcomes for fetuses in low-risk pregnancies [16,17]. However, in cases where FGR is diagnosed, ultrasound evaluation becomes essential to assess fetal growth, amniotic fluid volume, and umbilical artery Doppler blood flow. Ultrasound examinations should be performed every 2 weeks to monitor fetal growth dynamics and to differentiate between true FGR and constitutionally SGA fetuses.

In addition to biometric measurements, fetal Doppler studies and amniotic fluid assessments play a crucial role in refining the diagnosis of FGR and determining the optimal timing of delivery. Among these parameters has emerged as a valuable indicator of placental function and fetal well-being. Most studies have demonstrated that amniotic fluid assessment, usually by measuring the single deepest vertical pocket as an important adjunct in the monitoring and clinical decision-making process for pregnancies at risk of FGR. When combined with biometric and Doppler assessments, amniotic fluid evaluation can enhance the accuracy of diagnosis and help guide timely intervention to reduce adverse perinatal outcomes [12,18,19].

### 1.3. Doppler Surveillance in Fetal Growth Restriction

Doppler studies are vital for assessing fetal hemodynamics and placental function. Key parameters include Umbilical Artery Doppler (UA), Middle cerebral artery (MCA), Cerebroplacental ratio (CPR) and Ductus venosus (DV) Doppler. Fetal Doppler studies are routinely employed in such cases to evaluate fetal well-being. Research has shown that in physiological pregnancies, umbilical vascular resistance progressively decreases as term approaches, leading to a corresponding decline in the pulsatility index (PI) [20]. Assessment of the umbilical artery is recommended through the measurement of peak systolic velocity, frequency shift, end-diastolic frequency shift, and mean peak frequency shift, with consideration of the systolic-to-diastolic ratio, resistance index, and PI [21].

In cases of FGR resulting from placental insufficiency, umbilical artery impedance increases, often progressing to absent end-diastolic flow and eventually to reversed end-diastolic flow, as blood is preferentially redistributed toward the fetal brain. These hemodynamic changes lead to a secondary elevation in the PI [20,22]. Both absent and reversed end-diastolic flow in the umbilical artery are strongly associated with an increased risk of perinatal mortality. These findings serve as markers of fetal hypoxia, with studies demonstrating that clinical deterioration typically occurs within approximately 5 days in cases of absent end-diastolic flow, and within 2 days at most in fetuses exhibiting reversed end-diastolic flow [16].

Doppler velocimetry of the middle cerebral artery is also frequently utilized in current clinical practice. It is well established that cerebral vascular resistance decreases in cases of FGR, reflecting blood flow redistribution toward the brain at the expense of other organs. However, studies conducted by the Society for Maternal-Fetal Medicine have concluded that assessment of the middle cerebral artery is not essential in the evaluation of FGR, as it does not provide additional diagnostic value beyond that offered by umbilical vein Doppler velocimetry [8,22].

Low fetal cerebroplacental ratio measured in FGR cases within 72 h of delivery indicative for cerebral redistribution could identify pregnancies at risk of placental insufficiency likely to require cesarean section and obstetric intervention for intrapartum fetal compromise [23,24].

### 1.4. Maternal Perception of Fetal Movements

Maternal perception of fetal movements is also a good tool for monitoring fetal in pregnancies, including pregnancies with FGR or SGA [16]. Fetal movements are a non-invasive and relatively simple indicator of fetal health, as they reflect the fetus’s neurological and musculoskeletal integrity and can signal potential compromise. In the context of FGR, where placental insufficiency can lead to fetal hypoxia and growth disturbances, maternal perception of fetal movements becomes an important subjective measure that complements clinical surveillance and diagnostic tools like CTG and Doppler velocimetry.

Research indicates that changes in fetal movement patterns are often the first signs that mothers notice when fetal health is at risk [25]. A reduction in fetal movement, or even a complete absence, is commonly associated with fetal distress and may be an early indicator of hypoxic conditions. Several studies have highlighted that maternal perception of reduced fetal movements in pregnancies complicated by FGR is significantly correlated with adverse outcomes, including stillbirth and neonatal morbidity [26,27]. Notably, FGR fetuses are more likely to exhibit decreased movement in response to placental insufficiency and reduced oxygenation, which can result in alterations in fetal behavior and motor activity [28].

However, it is essential to recognize that maternal perception of fetal movements is subjective and influenced by various factors, including maternal anxiety, maternal obesity, and gestational age. In some cases, mothers may not perceive subtle changes in fetal movement, particularly in the early stages of FGR or in pregnancies where fetal movements are typically low [29]. Conversely, some women may report heightened sensitivity to fetal movements, which may lead to over-reporting of perceived fetal distress.

The utility of maternal perception of fetal movements as a screening tool is supported by several studies that have shown that women who notice a reduction in fetal movements often present for earlier clinical evaluations, leading to timely interventions [30]. For instance, interventions such as early delivery or increased fetal surveillance have been shown to improve outcomes when maternal concerns regarding fetal movements prompt more frequent monitoring [31].

In summary, encouraging women to be vigilant about changes in fetal movement and to seek medical advice promptly is an important strategy in the management of FGR.

### 1.5. Role and Interpretation of Cardiotocography

Cardiotocographic monitoring (electronic fetal heart rate, non-stress test) is an essential tool in contemporary obstetric practice, employed both during labor and in the antenatal period. By simultaneously analyzing fetal heart rate (FHR) and uterine contractile activity, CTG provides an indirect yet sensitive assessment of fetal well-being, particularly in the early detection of intrauterine hypoxia. The primary objective of CTG is to identify early signs of fetal compromise, thereby preventing irreversible deterioration in fetal status and guiding appropriate obstetric management.

Electronic FHR monitoring was introduced into clinical practice over six decades ago, initially met with limited acceptance due to the technical constraints of the time and skepticism within the medical community. Despite the publication of numerous studies over the years, there remains a lack of universally accepted protocols or clinically validated, standardized algorithms capable of replacing or unifying the human interpretation of CTG tracings.

Consequently, the effective use of CTG requires not only a solid understanding of fetal physiology and the mechanisms of adaptation to intrauterine stress, but also ongoing training of healthcare professionals to ensure accurate interpretation. This is crucial to maintaining an optimal balance between justified intervention and the avoidance of iatrogenic complications.

CTG remains one of the primary modalities for monitoring fetal well-being from approximately the 20th week of gestation through to delivery [32].

The majority of existing studies have been conducted during labor or near the time of delivery, when various coexisting conditions may contribute to or confound the assessment of fetal hypoxia [33,34,35,36,37].

#### 1.5.1. Standard Criteria and Interpretation of Normal Fetal Heart Rate in Cardiotocography

A reassuring CTG indicates a well-oxygenated fetus with no evidence of hypoxia or acidosis. According to guidelines such as those from NICE (National Institute for Health and Care Excellence) and FIGO (International Federation of Gynecology and Obstetrics), a CTG is classified as *reassuring* when all the following criteria are met [38]:

- Baseline Fetal Heart Rate: The baseline heart rate measured in beats per minute. The baseline rate should be between 110 and 160 beats per minute. This range reflects normal autonomic regulation and is considered physiologically stable.
- Baseline Variability: Variability refers to the fluctuations in the baseline FHR, reflecting the interplay between sympathetic and parasympathetic nervous systems. Reassuring variability lies within 5 to 25 beats per minute.
- Accelerations: Accelerations are transient increases in FHR below the baseline, typically by ≥15 bpm lasting for ≥15 s. While their presence is reassuring, their absence does not automatically indicate pathology if other parameters are normal.
- Decelerations: Are decrease in FHR below the baseline that lasted longer than 15 s and had an amplitude greater than 10 bpm. Early deceleration, meaning the start of the deceleration, is simultaneous with the start of uterine contraction, and the end of the deceleration end simultaneous with the contraction, and late decelerations means the start of the deceleration is after the start of the contraction and the end of the deceleration is after the end of the contraction. There should be no decelerations, or only early decelerations, which are typically benign and associated with fetal head compression during contractions. Variable or late decelerations may suggest cord compression or uteroplacental insufficiency and warrant further evaluation.

**Figure 1:**
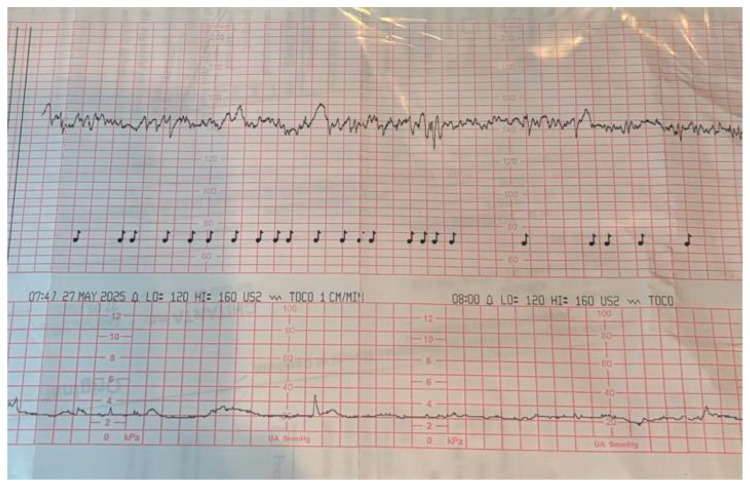
Cardiotocographic trace fulfilling normality criteria, suggesting fetal well-being. The recording shows a reactive pattern with normal baseline variability, the presence of accelerations, and the absence of pathological decelerations.

When all the above parameters are within the normal range, the CTG can be confidently interpreted as reassuring, suggesting that the fetus is not currently hypoxic. An example of such a CTG trace is shown in Figure 1. Continued surveillance is indicated, but no immediate intervention is required solely based on the CTG findings.

It is also important to consider that starting around 28 weeks of gestation, a healthy fetus alternates between periods of active and quiet sleep. During active sleep, the fetus shows accelerations, high variability in FHR, and clusters of movements. In contrast, quiet sleep features low FHR variability and fewer movements. The appearance of active sleep characteristics in a non-stress test (NST) primarily indicates fetal well-being. However, it is not possible to assess fetal health during quiet sleep, as the low FHR variability seen in healthy fetuses in this state cannot be distinguished from that of compromised fetuses. Therefore, monitoring should be continued long enough to observe at least one active sleep episode to differentiate between quiet sleep and a nonreactive NST reading [39].

#### 1.5.2. Standard Criteria and Interpretation of Normal Computerized Cardiotocography

Computerized cardiotocography is an advanced method of fetal surveillance that employs software algorithms to provide objective analysis of FHR patterns. We use Omniview-SisPorto Central—Fetal Monitoring Software Version 4.0.15. Most commonly used in antenatal care for high-risk pregnancies, cCTG improves reproducibility and minimizes observer subjectivity. The most widely used interpretative system is based on the Dawes–Redman criteria, which assess multiple parameters of FHR variability and reactivity [40].

In modern obstetric care, cCTG has become the preferred modality for fetal monitoring, particularly in the antenatal surveillance of high-risk pregnancies. This method is increasingly used due to its ability to provide a more objective, standardized, and time-efficient interpretation of the cardiotocographic trace, compared to traditional visual assessment.

By employing advanced analytical algorithms—most notably the Dawes–Redman criteria—cCTG evaluates FHR patterns and variability with high precision. This significantly reduces inter- and intra-observer variability, enhancing diagnostic consistency and aiding clinical decision-making.

The Dawes–Redman criteria are a set of objective, computerized parameters developed to assess fetal well-being using cCTG. These criteria are embedded in analysis software (e.g., Oxford Sonicaid systems) and are primarily used in antenatal monitoring, particularly in high-risk pregnancies beyond 28–32 weeks’ gestation [41,42].

They assess both FHR variability and reactivity, producing a pass/fail result based on whether all required conditions are met. A “pass” indicates low risk of fetal hypoxia at the time of recording.

A cCTG is considered normal (or *reactive*) when the following parameters are met [41,42]:

- Baseline Fetal Heart Rate: The baseline heart rate measured in beats per minute. A stable heart rate between 110 and 160 beats per minute is considered normal and reflects adequate autonomic control.
- Short-Term Variation (ST variation): Measure beat to beat variation (expressed in milliseconds) of fetal heart rate which cannot be interpreted by the human eye.
- Short-Term Variability (STV)**:** Measure beat to beat variation (expressed in bpm), sequential epoch-to-epoch variation.

The analysis segments each minute of the cCTG recording into sixteen epochs, each lasting 3.75 s. Within each epoch, the average FHR is computed, expressed both in beats per minute and as the corresponding pulse interval in milliseconds. The differences between successive pulse intervals are then calculated and averaged within each one-minute interval. These minute-level averages are subsequently averaged across the entire duration of the NST to derive the reported in milliseconds for ST variation. This is a key quantitative marker of fetal well-being, it is a predictor for fetal acid-base status and acidemia before labour. An ST variation greater than 3.0 milliseconds is considered reassuring. Low ST variation may be an early sign of fetal compromise, especially in the absence of fetal movements or accelerations. In healthy eutrophic fetuses ST variation increases from about 6 ms at 26 weeks to 8 ms at term.

- Long-term variability (LTV) measure minute-by-minute range of pulse intervals and represents the broader oscillations in baseline FHR over time. A normal LTV ranges between 5 and 25 bpm, indicating an intact autonomic nervous system and adequate fetal oxygenation. Abnormally low or high LTV may signal fetal hypoxia, sedation, or altered neurological function.
- Accelerations: The presence of at least one acceleration (a transient increase in FHR of ≥10 bpm lasting ≥15 s) indicates fetal reactivity. While not mandatory, accelerations enhance the reassuring nature of the trace.
- Episodes of High Variation: These are periods of increased FHR variability and are associated with fetal movement. A normal cCTG should include at least one episode of high variation during the monitoring period.
- Absence of Decelerations: A normal cCTG should show no late or prolonged decelerations. Occasional early or brief variable decelerations may be considered acceptable if the overall trace is otherwise normal.
- Fetal Movements (Optional): Detection of fetal movements during the trace is supportive but not essential for a normal classification under the Dawes–Redman system.

#### 1.5.3. Duration of Recording

**Figure 2:**
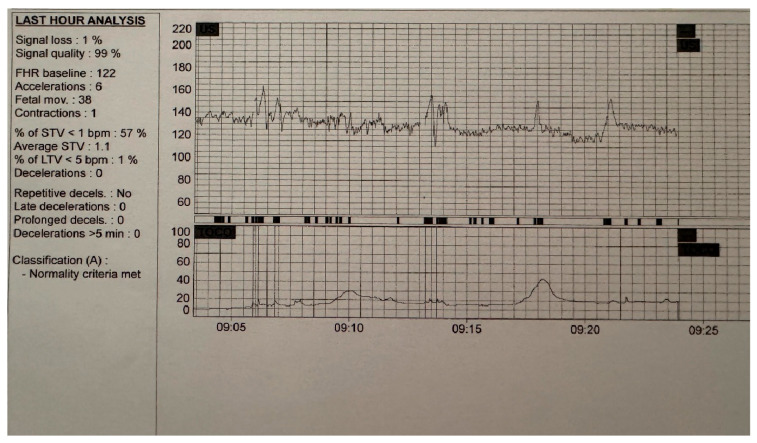
Computerized cardiotocographic trace classified as Category A—Normality Criteria Met, according to Dawes–Redman standards. The trace demonstrates parameters consistent with a reassuring fetal status, including appropriate short-term variability, presence of accelerations, and absence of pathological decelerations.

A typical cCTG recording lasts 20 to 60 min, with the system terminating automatically once all criteria for a normal trace have been satisfied. An example of such a case is shown in Figure 2. The figure presents a cCTG recording obtained using the Omniview Sis Porto Central-Fetal Monitoring Software Version 4.0.15, with an adequate duration of more than 20 min, fulfilling the criteria for classification as normal or reactive.

## 2. Materials and Methods

A comprehensive literature search was performed in PubMed and Web of Science covering the period between January 2001 and December 2024. Three predefined search strategies were employed using the terms <intrauterine growth restriction> AND < cardiotocography>, intrauterine growth restriction > AND <CTG>, <SGA> AND <CTG>. These keywords were selected to ensure that both the broader terminology of intrauterine growth restriction and the more specific small-for-gestational-age concept were captured, while also accounting for variations in the use of “CTG” and “cardiotocography” across the literature.

To refine the search results and improve relevance, database-specific filters were applied. In PubMed, the search was restricted to human studies and English-language publications. In Web of Science, results were limited to English language and the subject category Obstetrics & Gynecology, which allowed us to focus on clinically relevant obstetric research while excluding unrelated fields.

Following the initial retrieval of articles, duplicates were removed and all remaining studies were screened through both title/abstract and full-text review. **Inclusion criteria** comprised original research published in English, with an adequate sample size beyond case reports, and explicitly addressing the role of computerized cardiotocography (cCTG) in the context of fetal growth restriction (FGR/SGA). To ensure methodological consistency, several **exclusion criteria** were applied: review articles, twin pregnancies, studies assessing conventional CTG without computerized analysis, publications focused exclusively on cCTG or FGR without linking the two, research investigating cCTG in multiple pathologies without distinct FGR analysis, studies evaluating the effect of antenatal corticosteroid administration (e.g., betamethasone), and articles not available in full text.

It is important to note that a substantial proportion of eligible studies derived their cohorts from the TRUFFLE trial, a large multicenter study on early-onset FGR. These articles were retained when they presented original analyses or secondary outcomes directly related to the use of cCTG in the surveillance of FGR, as they provide high-quality data and remain central to the evidence base in this field.

As this is a narrative review, no formal meta-analysis was performed. Instead, the findings were qualitatively synthesized to identify recurrent patterns and highlight key methodological and clinical aspects. Particular attention was paid to differences between early- and late-onset FGR, the integration of STV with Doppler indices and the biophysical profile (BPP), and the development of novel computerized indices such as signal complexity measures (e.g., Lempel–Ziv Complexity, Multiscale Entropy), PRSA-derived parameters (AAC), and phase-rectified slopes (APRS/DPRS). These approaches were assessed in terms of their potential to enhance the diagnostic and prognostic value of cCTG in FGR.

This narrative review is part of a doctoral thesis and is based exclusively on previously published literature. As it does not involve original research with human or animal subjects, ethical approval was not required.

As part of the broader research conducted for the doctoral thesis, a discrepancy was noted between the normal values for STV described in the literature and those recorded by the cCTG systems used in our analysis (Omniwiev SisPOrto). Upon contacting the software manufacturer, it was clarified—through their kind support—that the system refers to short-term variability, whereas many scientific sources use the same abbreviation, “STV”, to denote short-term variation. This terminological overlap, although subtle, carries important implications for the interpretation of fetal monitoring data.

## 3. Results

A total of 17 studies and 5425 cases were identified as eligible and included in the final synthesis. This selection was considered sufficiently representative of the available literature, balancing methodological rigor with clinical relevance. The main characteristics, methodological approaches, and key findings of the included studies are summarized in Table 2, which provides a structured overview of the current evidence on computerized cardiotocography in the context of fetal growth restriction.

Recent studies consistently demonstrate that reduced short-term variability of the FHR remains a robust correlate of fetal compromise and adverse perinatal outcomes. Thresholds around 3.0 ms are generally considered concerning, while values near 2.6 ms are linked to high-risk situations and often indicate fetal acidemia or the need for prompt delivery. In early-onset FGR (before 32 weeks), DV Doppler abnormalities frequently precede or coincide with STV reductions; combining cCTG with DV Doppler improves delivery-timing decisions, as shown in randomized frameworks. In late-onset disease (from 32 weeks), STV alterations are subtler and require integration with the cerebroplacental ratio, middle cerebral artery Doppler, and time spent in high variability; advanced indices (e.g., acceleration and deceleration slopes, delta index) add discrimination when STV is borderline. Interpretation must consider gestational-age-specific reference ranges and fetal behavioral states (active versus quiet sleep), which meaningfully influence variability. Serial, trend-based assessment strengthens prognostic value relative to single measurements. Concomitant clinical factors—including antihypertensive exposure, maternal comorbidities, and placental insufficiency severity—modulate both STV and Doppler signals and should be explicitly accounted for. Despite convergent findings, heterogeneity in thresholds and outcome definitions, fragmented datasets with incomplete neonatal linkage, and limited standardization across devices constrain certainty. Overall, the literature supports STV as an objective adjunct to Doppler velocimetry and the BPP, with the greatest utility arising from integrated, longitudinal surveillance and the development of gestational-age-calibrated thresholds validated in large, outcome-linked cohorts.

### 3.1. Evidence from Recent Studies

A major challenge in studies on computerized fetal monitoring is the lack of comprehensive databases. A meaningful analysis or machine learning application requires a large dataset, which is currently lacking. Furthermore, the continuity of individual cases is often difficult to follow, as patients may be monitored in one center, seek emergency care in another, and ultimately deliver in a completely different facility. For example, one study included 25,000 CTG recordings collected between 2013 and 2021, but birth outcome data and information on fetal condition at delivery were available for only 20% of the women [43]. These are some of the reasons why building a sufficiently large and comprehensive database remains highly challenging, making it difficult to obtain robust and clinically valuable results.

Fetal growth restriction (FGR) most commonly arises in the context of an underdeveloped or dysfunctional placenta, which fails to provide an adequate supply of oxygen and nutrients to the fetus. This chronic hypoxic state activates the hypothalamic–pituitary–adrenal (HPA) axis and initiates a cascade of fetal compensatory mechanisms that impact FHR patterns. In the initial stages, heart rate variability remains preserved; however, as hypoxia progresses, classical signs of fetal compromise become evident, including:

- decreased baseline variability;
- absence of accelerations;
- the emergence of late decelerations;
- sustained bradycardia or tachycardia.

Hypoxemia and acidemia induce modifications in autonomic nervous system regulation, affecting both sympathetic and parasympathetic activity, which manifest as a reduction in FHR variability, particularly reflected by diminished STV on cCTG. The presence of late decelerations is indicative of a chemoreceptor-mediated response to fetal acidemia, as well as a direct depressant effect of acidemia on myocardial tissue integrity [44].

Antihypertensive treatment also may interfer with maternal hemodynamics, on cCTG accelerations and STV will be affected after Clonidine or other antihypertensive medication, probably by affecting placental perfusion [45,46].

The subsequent section presents a comprehensive review of recent literature (2014–2021) that has elucidated nuanced aspects of CTG and cCTG monitoring which may be specifically indicative in case of FGR. Furthermore, these studies examine correlations between CTG/cCTG patterns and other diagnostic modalities, with the goal of informing and enhancing the optimal management of affected fetuses. All of these data are summarized in Table 2.

**Table 2:** A synthesis of recent literature examining cardiotocographic parameters indicative of fetal growth restriction and their associations with complementary diagnostic modalities.

Author, Publication Year of the Study	Study Cohort	Results Related to Computerized Cardiotocography Alterations On Variability, STV, ST Variation and LTV	Correlation with Other Modalities of Surveillance/Management Implications
Hecher, K. 2001 [47]	110 singleton pregnancies with growth-restricted fetuses after 24 weeks (early and late FGR)	Low ST variation associated with imminent fetal compromise. ST Variation < 3 ms often indicated delivery within 5 days of absent/reversed end-diastolic flow.	Before 32 weeks of gestation, the PI of the DV and STVariation in FHR are crucial indicators for assessing the appropriate timing of delivery. If either parameter shows persistent abnormalities, delivery should be taken into consideration.
Anceschi, M.M. 2004 [48]	24 FGR pregnancies with abnormal Doppler velocimetry, monitored with cCTG before cesarean delivery	STV correlated significantly with umbilical artery blood gases (pH, pCO_2_). An STV threshold <4.5 ms predicted severe acidosis (pH <7.00) and hypercarbia (pCO_2_ >80 mmHg) with 100% sensitivity and 70–78% specificity. Other cCTG parameters showed no significant associations.	STV is a reliable marker of fetal acidosis and hypercarbia in FGR with Doppler abnormalities. A cutoff of 4.5 ms may serve as a clinical reference for timing delivery.
Soncini, E. 2006 [49]	50 FGR pregnancies, 186 cCTG recordings evaluated in conjunction with Doppler (UA, MCA, DV).	Absent/reversed end-diastolic flow in UA was linked with reduced STV, variability, and accelerations. Brain-sparing (MCA/UA <1) showed no association with cCTG changes. DV abnormalities were closely related to loss of variability, indicating advanced compromise. In milder cases, physiological maturation of cCTG with gestational age was present, but absent in severe FGR.	cCTG alterations parallel Doppler deterioration, with DV changes most predictive of advanced decompensation. Combined use of cCTG and Doppler (UA, DV) is essential for early recognition of fetal compromise in FGR.
Ferrario, M. 2009 [50]	59 fetuses at 27–34 weeks (17 normal, 19 non-severe FGR, 23 severe FGR)	Signal complexity indices (Lempel–Ziv Complexity, Multiscale Entropy) effectively discriminated severe FGR from both normal and non-severe FGR. Severe FGR showed reduced entropy/complexity, indicating loss of physiological autonomic control. A multiparametric approach (LZC + MSE slope) improved sensitivity and accuracy compared with single measures.	Complexity analysis of fetal heart rate provides early identification of severe FGR and differentiates it from non-severe cases. Multiparametric methods show promise for enhancing prenatal surveillance.
Huhn, E.A. 2011 [51]	74 FGR vs. 161 normal pregnancies, gestational age–matched (retrospective, single-center).	Both STV and PRSA-derived AAC were significantly reduced in FGR compared with controls. AAC demonstrated superior discriminatory capacity over STV, with more balanced sensitivity and specificity.	PRSA-derived AAC provides additional insight into fetal autonomic control and may be a more accurate marker of FGR than STV. Supports further longitudinal validation for outcome prediction.
Tagliaferri, S. 2015 [52]	120 pregnancies ≥30 weeks (59 normal, 61 IUGR), retrospective cross-sectional.	Phase-rectified slope indices (APRS/DPRS) discriminated IUGR from normal more accurately than conventional cCTG parameters (STV, LTI, entropy, Lempel–Ziv). Both indices showed significant correlation with cord blood pH. Predictive value was strongest before 34 weeks.	APRS and DPRS provide superior diagnostic performance over standard cCTG metrics, particularly in early-onset IUGR, and represent promising tools to optimize delivery timing.
Lobmaier, S. 2016 [53]	279 very preterm FGR fetuses (26–32 weeks), secondary analysis of TRUFFLE study	Applied phase-rectified signal averaging (PRSA: AAC, ADC) to raw cCTG data and compared with STV. AAC/ADC declined earlier (72 h before delivery) than STV (<48 h). PRSA indices predicted antenatal death and Apgar <7 more accurately than STV, but neither method predicted neurodevelopment at 2 yrs.	PRSA detects fetal deterioration earlier than STV and may provide superior short-term prognostic value in very preterm FGR.
Amorim-Costa, C. 2017 [54]	11 687 cases were analyzed retrospective, of which 1 986 were identified as small for gestational age (SGA) < p10, and 543 FGR < p3	SGA fetuses exhibited a lower average long-term variability (LTV), which was the parameter showing the most statistically significant difference, as well as reduced average STV and fewer accelerations. Baseline FHR was lower in the SGA group compared to normal fetuses starting from 34 weeks of gestation. The number of decelerations remained consistently similar between both groups.	Differences most evident between 28–35 weeks; deceleration frequency remained similar across groups.
Strolux, L. 2017 [55]	1163 FGR < 3 th centiles and 1163 control cases	FGR fetuses show a lower percentage of high variability (active sleep) compared to the normal population	Combining HRV markers with inferred fetal sleep states improved early-onset FGR detection.
Frusca, T. 2017 [56]	511 cases, TRUFFLE study (The Trial of Umbilical and Fetal Flow in Europe)	<32 weeks, DV Doppler abnormalities sometimes preceded STV reduction; waiting until cCTG became abnormal increased risk of fetal death or neurological issue. >32 weeks abnormal CTG parameters precede DV abnormalities	Delivery between 26–28^+6^ Weeks: - DV absent a wave associated with cCTG-STVariation <2.6 milliseconds at 26^+0^–28^+6^ weeks and <3 milliseconds at 29^+0^–31^+6^ weeks. -decelerations Delivery after 32 weeks: -Spontaneous repeated persistent deceleration -UA modifications ARED flow may always prompt delivery >32 weeks and AED >34 weeks.
Visser, G.H.A. 2017 [57]	310 pregnancies with early-onset FGR (<32 weeks) from the TRUFFLE trial, randomized to monitoring with cCTG-STV or DV Doppler.	Only one-third of deliveries followed the randomized assignment; the majority were triggered by safety-net criteria (recurrent decelerations, markedly reduced STV) or maternal indications. Intact survival at 2 years was higher in the DV groups (86%) compared with the cCTG group (77%, *p* = 0.049). When delivery was indicated directly by abnormal DV findings or reduced STV, intact survival rates were comparable (~80–88%).	Exclusive reliance on cCTG-STV, particularly when intervention was delayed until safety-net triggers occurred, was associated with poorer long-term outcomes. The best results were achieved with integrated monitoring (DV Doppler combined with cCTG), supporting a multiparametric approach to optimize delivery timing in early-onset FGR.
Wolf, H. 2017 [58]	149 cases, early FGR from TRUFFLE study	<32 weeks: there was no association of STVariation regression coefficients, a last low STVariation or/and recurrent decelerations with short or long term infant outcomes.	Delivery is indicated in case of DV absent a wave associated with low ST variation or recurrent decelerations.
Graupner, O. 2018 [59]	66 cases, late-onset SGA fetuses	No significant difference in STV median values between controls, SGA and FHR pregnancies	A higher proportion of late SGA fetuses had STV <5th percentile, despite nonsignificant median differences.
Baier, F. 2019 [60]	41 fetuses with early-onset sever FGR	STV stable until delivery day; sharp fall only at birth. UA/MCA Doppler showed continuous deterioration 3 weeks before delivery; DV/UT remained stable. Poor STV–Doppler correlation.	STV is a late marker; UA/MCA Doppler changes precede and should guide timing. Daily combined monitoring recommended.
Strumpfe, F. M. 2019 [61]	97 cases of SGA	Reduced STV on cCTG correlated with higher NICU admission risk.	Combination of abnormal Ductus venosus PI and low STV is more related to perinatal death compared to fetuses with a single abnormal parameter.
Esposito, G. 2021 [62]	95 FGR cases, 154 were included in the control group	Delta Index, Short-Term Variability, Long-Term Variability, and both Acceleration and Deceleration Phase Rectified Slopes (APRS and DPRS)—were found to be reduced	Integrating advanced cCTG parameters with Doppler improved identification of fetuses at risk for NICU admission.
Bruin, C. 2022 [63]	367 pregnancies with early-onset FGR (<32 wks), retrospective cohort (last 5 days before delivery/fetal death)	Compared PRSA (AAC/ADC) and STV, alone and in combination with recurrent decelerations. Both reduced PRSA and low STV (± decelerations) were associated with adverse perinatal condition; PRSA did not outperform STV. Combining either index with decelerations improved prediction (sensitivity between 80–90%, specificity 30–40%).	PRSA and STV are equivalent; strongest predictive value achieved when integrated with deceleration analysis. Single-parameter use is inadequate → supports multiparametric monitoring in early-onset FGR.

Short-term variation (STV); Long-term variability (LTV); Heart rate variability (HRV); Fetal heart rate (FHR); Fetal growth restriction (FGR); Umbilical artery (UA); Uterine artery (UtA/UT); Ductus venosus (DV); Absent End- Diastolic (AED); Absent or reversed end diastolic flow (ARED). Pulsatility index (PI); Computerized cardiotocography (cCTG); Neonatal intensive care unit (NICU); Acceleration/deceleration phase rectified slope (APRS/DPRS); Small for gestational age (SGA); Ductus venous pulsatility index (DVPI); Phase-Rectified Signal Averaging (PRSA); Average Acceleration Capacity (ADC); Lampel-Ziv Complexity (LZC); Multiscale Entropy (MSE).

According to a study from 2001 [47], the first alterations to appear are those in the umbilical artery PI, middle cerebral artery PI, and the amniotic fluid index. Using a 50% probability of an abnormal finding as a reference, an abnormally low STV typically appears about three weeks after abnormal waveforms first occur in the middle cerebral artery. The reduction in heart rate variability reflects decreased physiological fluctuations of the autonomic nervous system and reduced vagal modulation. Before 32 weeks of gestation, this reduction seems to coincide with the development of cardiac failure, as indicated by the opposing trends between abnormal DV flow patterns and STV abnormalities. However, this pattern does not necessarily apply to every individual fetus, as shown by the comparative analysis of the initial abnormal findings in the ductus venous pulsatility index (DVPI) and STV.

In this context, Anceschi et al. [48], prospectively evaluated 24 FGR pregnancies with Doppler velocimetry abnormalities, all delivered by cesarean section. cCTG recordings obtained within two hours before birth were compared with umbilical artery blood gases, showing that STV was significantly correlated with umbilical artery pH and pCO_2_. An STV threshold below 4.5 ms identified severe fetal acidemia (pH < 7.00) and hypercarbia (pCO_2_ > 80 mmHg) with high sensitivity and acceptable specificity, while other cCTG parameters showed no significant associations. The study concluded that STV is a reliable marker of impaired fetal oxygenation and acid–base status in FGR, and that a cutoff of 4.5 ms may serve as a clinically useful threshold to guide timing of delivery in cases with abnormal Doppler findings.

Similarly, Soncini et al. [49] explored the combined use of computerized CTG and Doppler velocimetry in FGR, emphasizing the complementary value of these modalities in detecting fetal deterioration. This longitudinal study evaluated 50 pregnancies complicated by FGR, with 186 cCTG tracings analyzed alongside Doppler velocimetry of the UA, MCA, and DV. The authors demonstrated that deterioration of UA Doppler indices, particularly absent or reversed end-diastolic flow, was strongly associated with reductions in STV, long-term variability, and accelerations. DV abnormalities were most closely correlated with severe loss of variability, indicating advanced fetal compromise, whereas brain-sparing (MCA/UA ratio <1) showed no consistent relationship with cCTG alterations. In milder cases, normal maturational increases in variability with advancing gestational age were observed, but this pattern was absent in severely compromised fetuses. The study highlighted the complementary role of cCTG and Doppler, with venous Doppler changes providing the most predictive insight when combined with cCTG deterioration.

Ferrario et al. [50] applied advanced heart rate variability analysis to 59 FHR tracings between 27 and 34 weeks of gestation, using nonlinear indices such as Lempel–Ziv Complexity (LZC) and Multiscale Entropy (MSE). Both measures clearly discriminated severe FGR from non-severe and normal fetuses, with reduced entropy and complexity reflecting impaired autonomic regulation. A multiparametric approach combining LZC with the MSE slope further improved diagnostic performance compared with single measures. These findings suggest that complexity analysis can facilitate earlier identification of severe FGR and strengthen antenatal surveillance strategies.

Huhn et al. [51] introduced a novel application of *phase-rectified signal averaging (PRSA)* to fetal heart rate analysis, comparing its performance to the established STV. In this retrospective study including 74 growth-restricted and 161 gestational age-matched normal fetuses, the PRSA-derived parameter *averaged acceleration capacity (AAC)* and STV were both significantly reduced in FGR cases. AAC demonstrated a higher discriminative capacity (area under the curve (AUC) 81%) compared with STV (AUC 70%), suggesting it may provide a more accurate assessment of autonomic regulation. The authors concluded that AAC offers additional insight into fetal compromise and holds promise as a complementary marker to STV, though longitudinal validation is required before clinical implementation.

Tagliaferri et al. [52] evaluated the diagnostic performance of *phase-rectified slope indices*—acceleration phase-rectified slope (APRS) and deceleration phase-rectified slope (DPRS)—in comparison with conventional computerized CTG parameters. In this retrospective cross-sectional study of 61 fetuses with FGR and 59 normal pregnancies beyond 30 weeks, both APRS and DPRS more accurately discriminated FGR from controls than STV, long-term irregularity, entropy, or Lempel–Ziv Complexity. Importantly, the indices showed significant correlations with cord blood pH, and their predictive value was strongest before 34 weeks’ gestation. The authors concluded that APRS and DPRS outperform standard cCTG parameters, particularly in early-onset FGR, and may represent valuable tools for refining delivery timing.

Building on earlier work with PRSA, Lobmaier [53] investigated whether PRSA-derived indices could provide earlier warning of fetal compromise in very preterm FGR. In this secondary analysis of 279 fetuses (26–32 weeks) from the TRUFFLE study, *average acceleration capacity (AAC)* and *average deceleration capacity (ADC)* were calculated from raw cCTG data and compared with STV. The authors found that AAC and ADC declined up to 72 h before delivery, whereas STV showed deterioration less than 48 h prior. PRSA indices predicted intrauterine death and low Apgar scores more accurately than STV, although neither method predicted long-term neurodevelopment at two years. The study suggested that PRSA may detect fetal deterioration earlier than conventional STV, offering greater short-term prognostic value in very preterm FGR.

In 2017, a study was published analyzing 11,687 fetuses and their cCTG recordings, of which 9,701 were from uncomplicated pregnancies and 1,986 from pregnancies complicated by small-for-gestational-age fetuses. The study compared cCTG variables between the two groups. Overall, no major differences were observed between the SGA and control groups. However, when the data were stratified by gestational age intervals, statistically significant differences emerged, with decelerations being the only variable that remained consistent across all gestational ages. SGA fetuses exhibited significantly lower average LTV —the parameter with the strongest statistical difference—along with reduced STV and fewer accelerations. These differences were most evident in the 28–35 weeks gestational age group. From 34 weeks onward, baseline FHR was also lower in SGA fetuses compared to those in normal pregnancies [54].

In 2017, a cCTG analysis was conducted on 1163 cases and an equal number of matched controls. The findings indicated that LTV during active sleep was superior to STV in distinguishing affected fetuses; however, the most predictive parameter was the number of minutes spent in high variation per hour, corresponding to active sleep. The study concluded that heart rate variability markers, combined with indirect information on fetal sleep states, can aid in the detection of early-onset FGR [55].

Perhaps the most well-known study in this field is the TRUFFLE study, which aimed to identify the optimal strategy for monitoring early-onset FGR while also evaluating neurodevelopmental outcomes up to two years of age. Conducted between 2005 and 2010 across 20 centers throughout Europe, the study sought to establish clear criteria for when immediate delivery is necessary and when it can safely be delayed for fetal benefit. Participants were divided into three study groups based on STV and DV Doppler values. The study clearly demonstrated that the best outcomes were achieved when both monitoring techniques—cCTG and Doppler—were used in combination. Another key conclusion was that in fetuses with early-onset growth restriction and abnormal DV Doppler findings, clinicians should not wait for CTG abnormalities to occur, as doing so significantly increases the risk of fetal compromise [56].

Within the framework of the TRUFFLE trial, Visser et al. [57] conducted a post hoc sub-analysis to clarify how actual delivery indications influenced long-term outcomes in early-onset FGR delivered before 32 weeks. Among 310 infants, only one-third were delivered strictly according to randomized monitoring strategies (cCTG-STV, early DV, or late DV changes), while the majority were delivered for safety-net criteria (mainly recurrent decelerations), maternal indications, or other fetal reasons. Intact survival at two years was higher in the DV groups (86%) than in the cCTG group (77%), with the poorest outcomes observed in fetuses delivered from the cCTG arm due to late-appearing decelerations. However, when delivery was based directly on abnormal DV or low STV, outcomes were comparable across groups (80–88%). The authors concluded that reliance on cCTG-STV alone, particularly when waiting for safety-net triggers, was associated with poorer outcomes, and that integrated longitudinal monitoring combining DV Doppler and cCTG offers the most effective strategy for optimizing delivery timing in early-onset FGR.

Another study based on data from the TRUFFLE cohort, including 149 cases, provided important refinement to the original study’s conclusions. It found that reduced STV and/or recurrent decelerations were not associated with adverse neonatal outcomes, suggesting that it may be safe to postpone delivery until these abnormalities appear—provided that the DVPI remains within the normal range [58].

In 2018, the first study evaluating the performance of STV on cCTG using Dawes–Redman criteria were published. Reduced STV values were observed primarily in severe cases of FGR, typically before 32 weeks of gestation. Although the study was conducted on a relatively small sample size, the results showed no significant differences in STV values among the control group, SGA and FHR abnormality pregnancies. However, a higher proportion of late-onset SGA fetuses had STV values below the 5th percentile compared to controls [59].

In an effort to clarify the longitudinal evolution of cCTG parameters in severe early-onset FGR, Baier et al. [60] conducted a retrospective analysis of 41 fetuses monitored between 24 and 34 weeks of gestation, yielding over 1,400 combined cCTG and Doppler observations. The study demonstrated that STV remained relatively stable throughout the surveillance period, showing a marked decline only on the day of delivery. By contrast, Doppler indices of the umbilical artery and middle cerebral artery exhibited continuous deterioration beginning approximately three weeks before delivery, while correlation between STV and Doppler parameters was poor. The authors concluded that STV alone does not provide a reliable longitudinal marker of deterioration and emphasized the need for at least daily cCTG monitoring in combination with Doppler, which demonstrated earlier and more consistent predictive changes.

A 2019 study analyzing cCTG parameters found that reduced STV was significantly associated with an increased risk of neonatal intensive care unit admission in term pregnancies affected by FGR. However, no significant associations were identified between these Doppler or cCTG parameters and umbilical artery pH ≤7.15 [61].

In 2021, a study was published based on a sample of 95 cases of FGR. The study demonstrated that pregnancies affected by late-onset FGR exhibited significantly altered cCTG parameters when compared to the control group. Specifically, there was a notable reduction in Delta Index, Short-Term Variability, Long-Term Variability, as well as in the Acceleration and Deceleration Phase Rectified Slopes (APRS and DPRS). These changes were statistically significant and indicative of compromised autonomic regulation in growth-restricted fetuses. Furthermore, the integration of these advanced cCTG metrics with Doppler velocimetry findings enhanced the ability to identify fetuses at increased risk for adverse perinatal outcomes, including those requiring neonatal intensive care unit admission [62].

In a more recent contribution, Bruin et al. [63] retrospectively analyzed 367 pregnancies complicated by early-onset FGR (<32 weeks) during the final five days before delivery or intrauterine death, comparing conventional STV with PRSA indices of AAC and ADC. Both reduced STV and low PRSA values were significantly associated with adverse perinatal condition, yet PRSA did not demonstrate superiority over STV. Importantly, predictive performance improved substantially when either measure was combined with the presence of recurrent decelerations, reaching sensitivities of 80–90% albeit at the cost of modest specificity. The study concluded that neither STV nor PRSA alone is sufficient and highlighted the value of multiparametric monitoring strategies for optimizing surveillance and timing of delivery in early-onset FGR.

### 3.2. Delivery Management Based on cCTG

Currently, the only available intervention for pregnancies complicated by FGR is delivery, which necessitates careful consideration of the optimal timing to balance the risks of iatrogenic morbidity against prolonged fetal exposure to a compromised intrauterine environment.

Moreover, compared with Doppler ultrasound, which requires specialized equipment and the expertise of maternal–fetal medicine specialists, cCTG is simpler, more cost-effective, and more widely accessible. This underscores the importance of clearly defining its role in the clinical management of FGR. In this context, the International Federation of Gynecology and Obstetrics (FIGO) has established evidence-based recommendations linking cardiotocographic findings with obstetric management strategies in pregnancies affected by FGR. These guidelines integrate gestational age with cCTG interpretation to balance the risks of prematurity against those of prolonged intrauterine exposure. Table 3 provides a structured overview of these recommendations, serving as a practical reference for clinicians in guiding the timing and mode of delivery.

The TRUFFLE study demonstrated that utilizing a combination of DV Doppler assessment, cCTG, and established safety criteria to guide delivery timing results in improved long-term neurodevelopmental outcomes for infants surviving beyond two years. To replicate the TRUFFLE trial’s success, it is essential to employ the same monitoring protocols and adhere to the criteria for labor induction, which are based on combined findings from DV Doppler and fetal electrocardiogram analysis [64].

In cases of late-onset FGR, there are currently no clear international guidelines regarding the optimal timing of labor induction [65,66].

However, experts recommend that in cases where the umbilical artery PI exceeds the 95th percentile, labor should be induced after 36 weeks of gestation, but no later than 37 weeks and 6 days [67].

Additionally, in cases with cerebral blood flow redistribution, delivery should be planned around 38 weeks of gestation, but no later than 38 weeks and 6 days. Vaginal delivery is not contraindicated when the umbilical artery Doppler is abnormal; however, continuous CTG monitoring during labor is required [67].

A delivery performed before 32 weeks or even 24 weeks incurs risks associated with premature birth, but a delay of delivery increases the risk of perinatal mortality and morbidity.

It is of particular interest to examine and compare international guidelines regarding the management of pregnancies complicated by FGR. Given the heterogeneity in definitions, diagnostic thresholds, and surveillance strategies, a comparative approach allows for a clearer understanding of both the consensus and variability among leading obstetric authorities. This comparison is especially relevant in clinical settings where guideline adaptation must consider local resources and patient populations.

The table below (Table 4) summarizes key aspects of six major guidelines (ACOG, RCOG, FIGO, SMFM, ISUOG, and SOGC), focusing on the definition of FGR, the role of Doppler ultrasound and cCTG, the utility of biomarkers, and the timing of delivery in both early- and late-onset cases [9,16,68,69,70,71]. Although there is general agreement on the use of umbilical artery Doppler and the clinical relevance of EFW below the 10th percentile, differences emerge in the interpretation of cerebroplacental ratios, the routine use of STV, and thresholds for intervention [9,16,68,69,70,71]. These distinctions highlight the importance of individualized care and the need for context-specific implementation of evidence-based recommendations.

## 4. Discussion

The use of cCTG has become increasingly central in the surveillance of pregnancies complicated by FGR. Its ability to quantify STV and other FHR characteristics provides clinicians with objective tools for assessing fetal autonomic regulation and detecting early signs of hypoxia. Numerous studies, including the TRUFFLE trial, have demonstrated that STV, especially when combined with DV Doppler indices, can guide the timing of delivery and improve perinatal outcomes, particularly in early-onset FGR.

Nevertheless, important limitations of CTG—both conventional and computerized—must be acknowledged. One of the primary concerns is the subjectivity and variability in interpretation, especially in conventional CTG, which often leads to inconsistent clinical decisions. Although cCTG reduces observer bias by providing numerical data, there is still a lack of universally accepted thresholds for many parameters, including STV, particularly across different gestational ages. This ambiguity can result in either premature delivery due to overestimation of fetal risk or delayed intervention when fetal compromise is underestimated.

Another limitation is the influence of fetal behavioral states on heart rate variability. For instance, fetuses in quiet sleep naturally exhibit lower STV values, which can be misinterpreted as pathological. Without considering the sleep–wake cycle—often indirectly inferred from high-variation episodes—the reliability of a single STV measurement is limited. Advanced indices like the Delta Index or the duration of high variability per hour have been proposed to refine the interpretation, but these are not yet widely integrated into clinical practice.

Gestational age also modulates CTG interpretation, with early gestation tracings typically showing reduced variability and fewer accelerations, even in healthy fetuses. This developmental variability complicates the establishment of fixed cut-off values and calls for gestational-age-specific reference ranges.

Another significant challenge is the lack of data continuity in real-world settings. Many patients are monitored in fragmented care systems, making it difficult to establish trends over time. The absence of complete datasets—including delivery outcomes—limits the ability to fully validate CTG-based prediction models. This is particularly relevant for emerging AI-based systems, which require large, annotated datasets for training and validation.

Furthermore, CTG reflects only the functional response of the FHR to intrauterine conditions, but does not offer direct information about fetal oxygenation, acid-base status, or placental function. Thus, it must be interpreted alongside other modalities, such as Doppler velocimetry, amniotic fluid assessment, and fetal biometry, for a more accurate and comprehensive picture.

Despite these limitations, when used appropriately and in conjunction with other diagnostic tools, CTG remains a valuable component of fetal surveillance. Its strength lies in serial assessments and trend analysis rather than isolated values. Continued research is needed to improve standardization, incorporate more advanced analytic tools, and develop integrated decision-support systems that minimize subjectivity and enhance clinical decision-making in the management of FGR.

## 5. Future Directions

Future research on FGR and CTG surveillance should focus on the development of standardized, gestational age-specific thresholds for STVariation and other cCTG parameters. Current variability in definitions and interpretation limits the reproducibility and applicability of findings across different centers. The creation of large, outcome-linked, multicenter databases is essential to validate predictive models and to enable the integration of advanced analytics, including artificial intelligence and machine learning, into clinical decision-making.

Moreover, future studies should investigate the longitudinal assessment of STVariation trends rather than isolated values, in order to improve early detection of subtle fetal compromise, particularly in late-onset FGR. Combining cCTG with multimodal surveillance—such as Doppler velocimetry, BPP, and maternal biomarker assessment—may enhance risk stratification and support individualized management strategies.

Ultimately, the goal of future research is to develop automated, evidence-based decision-support tools that integrate cCTG metrics, Doppler findings, and clinical data to optimize delivery timing, minimize the risks of prematurity, and reduce perinatal morbidity and mortality in pregnancies complicated by FGR.

## 6. Conclusions

Effective management of pregnancies complicated by FGR necessitates meticulous fetal surveillance and judicious determination of the optimal timing for delivery. cCTG remains a critical tool in assessing fetal well-being, offering valuable insights into fetal heart rate variability and the fetal response to chronic or acute hypoxia. In the context of FGR, however, CTG interpretation requires clinical expertise and precision, and must be correlated with adjunctive investigations such as Doppler velocimetry and fetal biometry to enhance its diagnostic value. Among the cCTG parameters, STV has proven to be a sensitive marker for detecting fetal compromise, particularly in early-onset FGR.

Furthermore, cCTG represents a less expensive tool both in terms of equipment and human resources than other investigations. Ultrasound machines are considerably more costly than cCTG devices, and Doppler assessment requires the expertise of a maternal–fetal medicine specialist to acquire and interpret vascular indices. By contrast, cCTG can be performed by trained midwives or nurses with automated analysis. Its value in FGR management should therefore be clearly established, and large multicenter studies together with international collaborative analyses are needed to standardize definitions and thresholds, harmonize clinical practice, and ensure comparability of data across different healthcare systems.

However, relying solely on STV interpretation is insufficient, as it must be integrated with other diagnostic data to guide clinical decision-making. Studies, such as the TRUFFLE trial, highlight the importance of combining cCTG findings with Doppler evaluations, especially the DVPI, to significantly improve decision-making on the timing of delivery, thus reducing the risks of unnecessary early intervention or delayed delivery that could lead to irreversible fetal injury. In late-onset FGR, while STV changes may be less prominent, advanced indices such as the Delta Index and acceleration/deceleration slopes provide further insight into autonomic regulation and fetal well-being.

Moreover, recent research on PRSA has shown that derived indices, including AAC, ADC, and phase-rectified slopes (APRS/DPRS), consistently outperform conventional cCTG parameters in discriminating FGR and can reveal signs of fetal deterioration earlier, particularly before 34 weeks of gestation. Evidence from both single-center investigations and secondary analyses of the TRUFFLE cohort suggests that the integration of these indices with established parameters such as STV and deceleration analysis enhances predictive accuracy. These findings reinforce the concept that no single parameter is sufficient, and that a multiparametric framework combining conventional cCTG, Doppler velocimetry, and advanced signal analysis represents the most robust strategy for optimizing prenatal surveillance and guiding delivery timing in early-onset FGR.

The interpretation of CTG patterns in FGR also requires consideration of factors like gestational age, fetal behavioral states, and the chronicity of placental insufficiency. Clinical experience plays a pivotal role in distinguishing between pathological tracings and physiological variants, particularly in borderline cases. Given the subjectivity of CTG interpretation and the absence of standardized criteria, there is a growing need for uniform protocols and the incorporation of artificial intelligence-based systems to improve diagnostic precision and enhance early detection of fetal compromise.

Ultimately, optimal management of FGR pregnancies depends on an integrated, individualized approach that synthesizes clinical, Doppler, and CTG data. This approach helps to strike a careful balance between the risks associated with prematurity and the dangers of intrauterine compromise, aiming to improve perinatal outcomes through continuous fetal surveillance and multidisciplinary decision-making.

## Figures and Tables

**Table 1:** Consensus-based definitions for early and late fetal growth restriction in singleton [7].

Early-Onset FGR (<32 Weeks)	Late-Onset FGR (≥32 Weeks)
EFW or AC <3rd percentile	EFW or AC <3rd percentile
UA with AREDV	≥2 of the following 3 criteria: EFW or AC <10th percentile EFW or AC crossing percentiles >2 quartiles on growth percentiles CPR <5th percentile or UA PI >95th percentile
EFW or AC <10th percentile, combined with one or more of the following: UA PI >95th percentile UtA PI >95th percentile	growth failure (drop in EFW or AC centile) or abnormal fetal Dopplers

Fetal abdominal circumference (AC); absent or reversed end-diastolic velocity (AREDV); cerebroplacental ratio (CPR); estimated fetal weight (EFW); pulsatility index (PI); umbilical artery (UA); uterine artery Doppler (UtA).

**Table 3:** Criteria for Delivery and Management Strategies by Gestational Age in Pregnancies Complicated by Suspected Fetal growth restriction Based on FIGO Guidelines on Intrapartum Fetal Monitoring for fetuses with Fetal Growth Restriction [16].

Gestational Age (weeks)	24–27 (Periviability)	28–30 (Early Viability)	31–33 (Viability)	34–36 (Late Preterm)	≥37 (Term)
**Absolute Indications for Delivery** (regardless of GA)	Severe maternal condition (e.g., pre-eclampsia) BPP score < 4/10 Sinusoidal pattern Repetitive late decelerations Absent baseline variability **cCTG STVariation < 2.6 ms**	Severe maternal condition (e.g., pre-eclampsia) Sinusoidal pattern or repetitive late decelerations Absent baseline variability BPP score < 4/10 **cCTG STVariation < 2.6 ms**	Severe maternal condition (e.g., pre-eclampsia) Sinusoidal pattern or repetitive late decelerations Absent baseline variability BPP score < 4/10 **cCTG STVariation < 2.6 ms**	Severe maternal condition (e.g., pre-eclampsia) Sinusoidal pattern or repetitive late decelerations Absent baseline variability BPP score < 4/10 **cCTG STVariation< 2.6 ms**	Severe maternal condition (e.g., pre-eclampsia) Sinusoidal pattern or repetitive late decelerations Absent baseline variability BPP score < 4/10 **cCTG STVariation < 2.6 ms**
**Relative Indications for Delivery** (GA-adjusted)	-	Absent or reversed DV a-wave BPP score < 6/10 **cCTG STVariation < 3.0 ms**	Reversed umbilical artery end-diastolic flow **cCTG STVariation < 3.5 ms**	Absent umbilical artery end-diastolic flow **cCTG STVariation < 4.5 ms**	Elevated umbilical artery PI
**Management Strategy**	Individualized decision based on maternal status and fetal condition if **EFW > 500 g**	Maternal monitoring and Doppler of the umbilical artery and DV, with NST or cCTG and BPP	Maternal and fetal surveillance including Doppler of umbilical and middle cerebral arteries (± DV), NST/cCTG and BPP	Maternal and fetal surveillance including Doppler of umbilical and middle cerebral arteries (± DV), NST/cCTG and BPP	Delivery may be indicated if **EFW < 10th percentile**, regardless of Doppler results

Biophysical Profile (BPP); Computerized Cardiotocography (**cCTG**); **Ductus Venosus (DV)**; Short-Term Variation (**STV**); Estimated Fetal Weight (**EFW**); Pulsatility Index (**PI**); Non-Stress Test (**NST**).

**Table 4:** Comparison of International Guidelines for the Management of Fetal growth restriction (FGR).

Aspect	ACOG (2021) [9]	RCOG (2022) [68]	FIGO (2015) [16]	SMFM (2020) [69]	ISUOG (2019) [70]	SOGC (2020) [71]
Definition of FGR	EFW <10th percentile confirmed by ultrasound; differentiation between early and late onset	EFW or AC <10th percentile; severe if EFW <3rd percentile or abnormal Doppler	EFW <3rd percentile or EFW/AC <10th percentile + abnormal Doppler	EFW <10th percentile, reduced growth velocity, or Doppler abnormalities	EFW <10th percentile or declining growth trajectory with abnormal Doppler	EFW <10th percentile or AC <10th percentile with additional risk factors
Use of Doppler Ultrasound	Recommended for all suspected FGR cases: UA Doppler primary; consider MCA and DV	Mandatory: UA for all; MCA and CPR/UCR for late-onset; DV for early-onset	Central to diagnosis: UA, MCA, CPR and DV if <32 weeks	UA and MCA in all; DV for early-onset or abnormal UA	Recommended: UA first-line; MCA, CPR and DV for fetal compromise	Routine use of UA; other vessels based on clinical context
Role of cCTG/STV	Optional; used if BPP inconclusive or in high-risk cases	Not routine; used only with abnormal Doppler or clinical signs	Recommended adjunct in early-onset or high-risk pregnancies	Preferred in late-onset FGR with reduced variability or absent accelerations	Useful for assessing fetal well-being when other parameters borderline	Considered in tertiary settings with access to computerized systems
Biophysical Profile (BPP)	Used alongside NST or cCTG to evaluate fetal well-being	Accepted, especially in late preterm and term FGR	Complementary to Doppler and CTG in early-onset	Used to aid timing of delivery with cCTG and Doppler	Recommended only in selected cases; not primary tool	Supportive tool, especially when Doppler or CTG uncertain
Biomarkers (PlGF/sFlt-1)	Not routinely recommended	Considered if preeclampsia suspected	Under evaluation for clinical utility	Optional if preeclampsia or placental dysfunction suspected	Not used routinely; research use	Not standard but discussed in placental dysfunction
Timing of Delivery (Early-onset FGR)	<32 weeks if reversed end-diastolic flow or DV abnormalities; 32–34 weeks for absent EDF	Delivery at <32 weeks for absent/reversed EDF or DV changes	<32 weeks if reversed EDF or DV abnormal; steroids recommended	Individualized; typically <32 weeks for reversed EDF or abnormal DV	Generally deliver <32 weeks for DV abnormalities or reversed flow	Planned delivery at <32 weeks in presence of significant Doppler abnormalities
Timing of Delivery (Late-onset FGR)	Delivery from 37 weeks if isolated FGR; earlier if Doppler abnormal	36–37 weeks if normal Doppler; earlier if CPR <5th percentile or abnormal CTG	From 37 weeks unless Doppler abnormal or STV reduced	36–38 weeks depending on fetal condition and Doppler	At 37 weeks or earlier with abnormal MCA/CPR	Around 37 weeks unless compromise indicated earlier

Fetal abdominal circumference (AC); Absent or reversed end-diastolic velocity (AREDV); Cerebroplacental ratio (CPR); Estimated fetal weight (EFW); Pulsatility index (PI); Umbilical artery (UA); Uterine artery Doppler (UtA).

## Data Availability

No new data were created or analyzed in this study. Data sharing is not applicable to this article.

## Abbreviations

The following abbreviations are used in this manuscript:

AAC	Average Acceleration Capacity
ACOG	American College of Obstetricians and Gynecologists
ADC	Average Deceleration Capacity
AED	Absent End- Diastolic
AFI	Amniotic Fluid Index
APRS	Acceleration phase-rectified slope
ARED	Absent or Reversed End-Diastolic flow
AUC	Area under the Curve
BPP	Biophysical Profile
cCTG	Computerized Cardiotocography
CPR	Cerebroplacental Ratio
CTG	Cardiotocography
DPRS	Deceleration phase-rectified slope
DV	Ductus Venosus
DVPI	Ductus Venosus Pulsatility Index
EFW	Estimated Fetal Weight
FIGO	International Federation of Gynecology and Obstetrics
FGR	Fetal Growth Restriction
FHR	Fetal Heart Rate
IGF-1	Insulin-like Growth Factor I
IGF-II	Insulin-like Growth Factor II
IGFBP-2	Insulin-like Growth Factor Binding Protein 2
IGFBP-3	Insulin-like Growth Factor Binding Protein 3
IGFBP-4	Insulin-like Growth Factor Binding Protein 4
IUGR	Intrauterine Growth Restriction
ISUOG	International Society of Ultrasound in Obstetrics and Gynecology
LTV	Long-Term Variability
LZC	Lempel–Ziv Complexity (signal complexity index)
MCA	Middle Cerebral Artery
MSE	Multiscale Entropy (multi-scale signal entropy index)
NICU	Neonatal Intensive Care Unit
NST	Non-Stress Test
PAPP-A	Pregnancy-Associated Plasma Protein-A
PI	Pulsatility Index
PRSA	Phase-Rectified Signal Averaging
RCOG	Royal College of Obstetricians and Gynaecologists
SGA	Small for Gestational Age
SMFM	Society for Maternal-Fetal Medicine
SOGC	Society of Obstetricians and Gynaecologists of Canada
STV	Short-Term Variation
TRUFFLE	Trial of Randomized Umbilical and Fetal Flow in Europe
UA	Umbilical Artery
UtA/UT	Uterine Artery
VIP	Vasoactive Intestinal Peptide
